# A Machine Vision—Based Pipe Leakage Detection System for Automated Power Plant Maintenance

**DOI:** 10.3390/s22041588

**Published:** 2022-02-18

**Authors:** Nengsheng Bao, Yuchen Fan, Zihao Ye, Alessandro Simeone

**Affiliations:** 1 Department of Mechanical Engineering, Shantou University, 243 Daxue Road, Shantou 515063, China; nsbao@stu.edu.cn (N.B.); 20ycfan@stu.edu.cn (Y.F.); zhye2@stu.edu.cn (Z.Y.); 2 Intelligent Manufacturing Key Laboratory of Ministry of Education, Shantou University, 243 Daxue Road, Shantou 515063, China; 3 Department of Management and Production Engineering, Politecnico di Torino, Corso Duca degli Abruzzi 24, 10129 Turin, Italy

**Keywords:** maintenance, automation, system monitoring, vision system, image processing

## Abstract

Industrial pipework maintenance inspection can be automated through machine vision-based effusion monitoring. However, colorless effusions such as water can be difficult to detect in a complex industrial environment due to weak illumination and poor visibility of the background. This paper deploys the reflective characteristics of effusion and its lower temperature compared to the environment in order to develop an automatic inspection system for power plant pipeworks’ maintenance. Such a system is aimed at detecting the colorless fluid effusion based on dual source images and a contour features algorithm. In this respect, a visible light source unit highlights the reflective features of the effusion edge. Meanwhile, high-definition images of the potential effusion are acquired under both visible and infrared lights. A customized image processing procedure extracts the potential effusion features from the infrared image to retrieve the region of interest for segmentation purposes and transfer such information to the visible light image to determine the effusion contour. Finally, a decision-making support tool based on the image contour closure is enabled for classification purposes. The implementation of the proposed system is tested on a real industrial environment. Experimental results show a classification accuracy up to 99%, demonstrating excellent suitability in meeting industrial requirements.

## 1. Introduction

Pipes are currently considered to be the safest and most cost-effective means of fluid conveyance in factories, especially for dangerous flammable fluids. However, liquid leakage is an inevitable problem in pipeline systems as a result of wear, corrosion, and damage of pipeline infrastructure [1]. Liquid leakage monitoring and detection is the primary task of many enterprises and governments within their occupational health and safety schemes [2]. This is due to the fact that liquid leakage not only causes huge economic losses every year but also brings potential dangers to the safe and stable operation of equipment. With reference to power plants, when liquid leakage occurs in the cooling apparatus, the stator windings are heated unevenly, causing an uneven heating of the iron core and anomalous vibration of the generator (resulting in a potential threat to the safe and stable operation of the generator set) [3]. The main reasons for pipeline leaks include the natural wear process of wear, corrosion phenomena on both the inner and outer pipe surface, damages due to excessive mechanical loads, assembling faults, thermal fatigue, and material defects [4]. The pipework failure is generally attributed to the ageing infrastructure and/or severe environmental conditions [5].

As a consequence, pipeline leakage is very likely to have an impact on the availability and productivity of the plants resulting in economic losses [6], health and safety hazards, and environmental damage [7,8]. Such factors highlight how it is necessary to improve the maintenance inspection capabilities by carrying out fluid leakage detection to ensure the reliability and safety of the equipment [9].

In such a context, this paper investigates a framework for an implementation procedure and experimental validation to automate maintenance tasks by identifying pipes defects such as leaking through a combined use of multi spectra imaging. This approach aims at detecting pipe leakages through the automatic recognition and characterization of water effusions utilizing a tailored hardware and image processing.

The remainder of this paper is organized as follows. A literature review is carried out exploring state of the art of the main technologies adopted in leaking detection and maintenance automation along with the scientific and technological challenges. The experimental setup utilized to collect digital images of water effusion in a powerplant environment is reported. A procedure for the image processing is described with the aim of extracting significant features of the effusion contour. A classification-based decision-making support system is then illustrated. The proposed system is finally implemented to inspect a number of real power plant pieces of equipment, and the results are discussed in terms of accuracy and industrial suitability.

## 2. State of the Art

The current literature and industrial practice surveys show that most of the advanced contributions are concentrated in three key enabling technologies, namely sensor monitoring, machine learning, and computer vision.

As regards the use of sensors and sensor networks in leakage detection, the variety of devices ranges from water meters for flow rate measurement [10] to pressure sensors [11] which allow for identification and localization of leakages in pipeworks. Application of tailored sensors can be found in [12], using a technology based on the short-circuit principle to identify leakages from different liquids and quantify their rate. C.M. Giorgio Bort et al. designed a real network leakage detection and location method based on pressure changes of pipeline pressure sensors. Alberto Martini et al. used a hydrophone and two accelerometers to monitor the vibration and acoustic phenomena related to the leakage flow to detect leakages in buried experimental facilities [13]. Wu Changrui et al. used laser scanning technology (based on the difference in the reflection intensity between the seepage area and the background area) to detect the area and location of the leakage water with 92% accuracy. However, due to the interference of cables and protective materials in the tunnel, great misjudgment could be caused [14]. Fabbiano et al. proposed a vibration analysis method, which used laser Doppler vibrometer to detect pipeline vibration parameters at fixed points, and determined whether there was a fluid leak by analyzing the change of vibration energy transmitted to the pipe wall [15]. Solomon Seyoum et al. proposed a sound analysis method in which non-invasive sensors were used to collect the sound signals of water-using equipment and the noise generated by water leakage was extracted for water leakage analysis and assessment [16].

Intelligent decision-making systems on pipe leakage assessment has been successfully carried out by applying machine learning algorithms. In this respect, Abdulla and Herzallah [17] used water supply pipeline flow rate, pressure data, and an artificial neural network (ANN) to configure the leak detection as a classification task yielding excellent accuracy results. Roya A. Cody et al. collected actual acoustic monitoring data and carried out anomaly detection through semi-supervised learning reaching a leakage detection rate up to 97.2% [18]. Alternative machine learning paradigms have been successfully implemented by Matsubara et al. that extracted time domain features from flow, pressure, and temperature in correspondence of pipeline inlet and outlet to setup a k-Nearest Neighbor (KNN) classification system for a binary pipeline leakage assessment [19]. Hong-Wei Huang et al. developed a deep-learning based leakage detection system endowed with a full convolutional neural network to performed semantic segmentation on the leakage features for metro shield tunnels structural safety monitoring and maintenance. [20]. Yang Liu et al. put forward a leakage detection method utilizing wireless sensor networks and features extraction via intrinsic mode function, approximate entropy, and principal component analysis (PCA) in order to setup a classification tool based on support vector machine (SVM) [21]. Jiheon Kang et al. proposed a water leakage detection and localization system utilizing a one-dimensional convolutional neural network (CNN) in conjunction with SVM (1D-CNN-SVM) improving the performance using a graph-based local search algorithm [22]. Salman Khalid et al. adopt machine learning to support the sensor selection in waterwall tube leakage detection for steam power plants [23].

Recent developments in visual detection systems involve the use of high-definition under fully visible light and infrared for the detection of leakage. In order to meet the needs of industry 4.0, Durga Prasad Penumuru et al. proposed an automatic material identification methodology based on machine vision and machine learning technology, which can successfully identify four different materials on the surface of a machine in the actual industry [24]. Je-Kang Park et al. used a convolution neural network (CNN) to directly recognize patterns without manual feature extraction [25].

As regards computer vision, the primary problem to address is the gap between high level concepts and low-level visual cues. In this context, illumination is one of the most effective factors in visual detection [26]. High-definition imaging allows for the use of the reflective characteristics of water masses for inspection purposes. In the field environment, when there is sufficient sunlight on the opposite side of the water mass, Chen Tianding et al. proposed a method aiming at the polarization characteristics of the water mass after reflection, using a CCD camera with a linear polarizing mirror to obtain images of water, and realize the segmentation and recognition of water features in the HCI space [27]. Concerning the light sources, light emission diodes (LED) represent a largely employed and cost-effective solution. Such technology results relatively narrow in terms of emission bandwidth, however it is characterized by a low spatial coherence, which reduces the generation of speckle-noise [28], indicating good suitability for image processing purposes in industrial monitoring scopes.

Infrared imaging is often used for effusion detection since the temperature of effusion is usually lower than that of the surrounding environment. Relevant research contributions, ranging from civil to industrial engineering applications, involve the use of a single infrared camera to detect the leakage areas in buildings due to infiltration deploying the temperature difference [29]. Terumi Inagaki et al. detected leakage through infrared thermal imaging technology according to the abnormal temperature field of the leakage point with lower temperature than the surrounding environment [30]. Ahmed Aatef et al. proposed a set of methods for comprehensive detection of water pipe leakage based on the characteristics of ground penetrating radar (GPR) images and infrared images, which achieved good results in estimating the area of liquid accumulation (the error is only 2.9–5.6%) [31]. Mohamed Fahmy et al. proposed a method of detecting pipeline leakage through the use of infrared sensors, which can successfully detect and locate the location of leakage when the temperature is appropriate [32].

Waterwall boiler tube leakage is the most frequent cause of failure in thermal power plants [23]. One specific study has shown a strong correlation between internal cracks and leakage phenomena, with the development of cavity nucleation and growth on grain boundaries eventually yielding macro-intergranular cracks [33].

**Figure 1:**
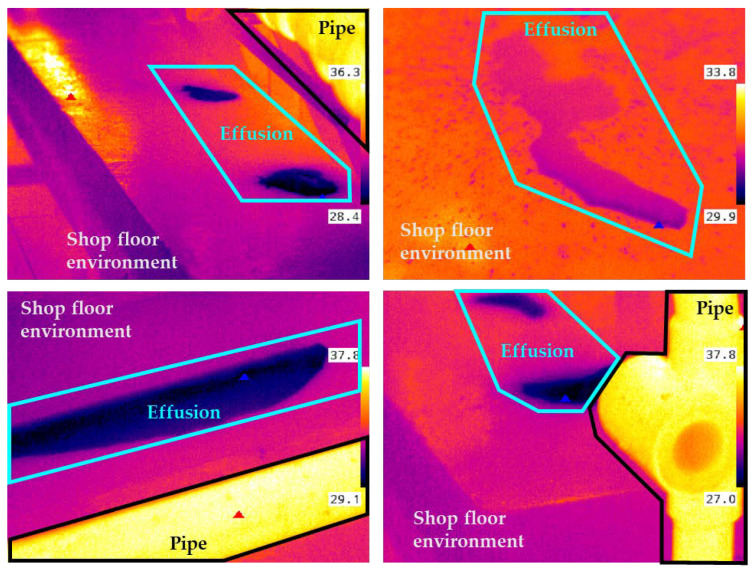
Infrared measurements (in °C) carried out at HUANENG thermal power plant. The variability range in effusion temperature is due to ambient temperature, different pieces of equipment and distance from pipe to the effusion.

In order to characterize the operating conditions, an industrial survey of temperature measurements was carried out at HUANENG power plant, Shantou, China, with the aim of defining the temperature ranges of the equipment to be inspected. Figure 1 shows four survey image instances in which the temperature of the working pipe ranges between 33 and 37.8 °C, the effusion on the ground ranges between 28 and 30 °C, and the ambient temperature is between 31 and 33 °C. The temperature survey was carried out for a large amount of equipment within the HUANENG facilities throughout a long period of time, showing that such temperature ranges are consistent in space and time with a very limited variability range (as shown in Figure 1).

**Figure 2:**
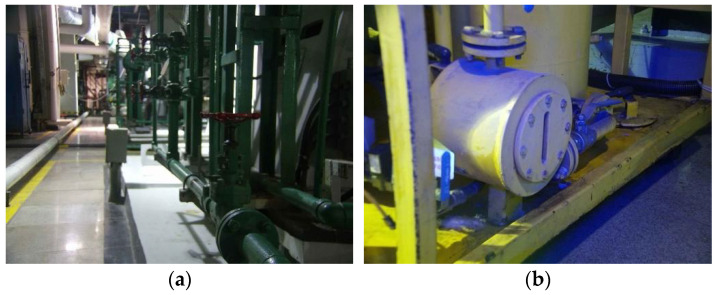
Significant difficulties in the actual factory environment: (**a**) Complex pipework layout; (**b**) diverse illumination conditions and non-uniform light distribution result in highly variable detection area background.

The visual detection technology survey suggests that the two major characteristics of colorless effusion, namely the reflective characteristic under full lighting and the low temperature characteristics in the environment, can be deployed by existing technology for detection purposes. However, the design and implementation of a comprehensive detection system results difficult in complex industrial environments. With reference to a generic thermal power plant, on the one hand, the site environment is complex and characterized by crisscrossing pipelines, with significant environmental interference problems occurring during maintenance inspection such as humidity and cold areas (as shown in Figure 2a). On the other hand, the areas affected by fluid effusion are usually poorly illuminated, making the colorless effusion detection hard to be directly performed (as shown in Figure 2b).

**Figure 3:**
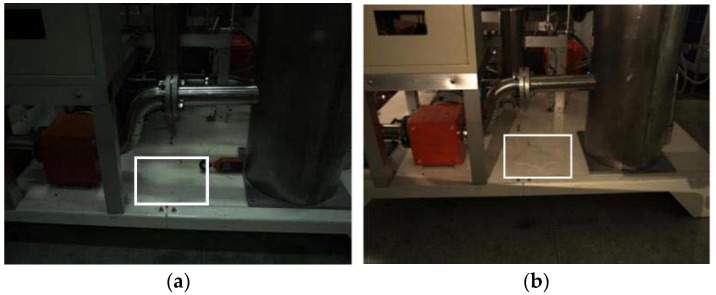
Difficult to detect colorless effusion (in the white box): (**a**) effusion feature in natural light; (**b**) effusion feature under LED flash light.

In general, the illumination conditions of the overall environment can be enhanced through a flash light, but visual characteristics of the leakage would still tend to appear as colorless, as shown in the white box mark in Figure 3a. The use of additional LED lights could enhance the water leakage edge features visualization, but the overall edge contour would look still fuzzy and hard to detect (as shown in Figure 3b).

This state-of-the-art review has highlighted the scientific and technological challenges in a machine vision-based pipe leakage detection. In addition to these, the operational complexity within an actual industrial plant such as the presence of steam, debris, complicated pipework, and safety hazards define the constraints for this research work.

Specifically, a robust approach aimed at the maintenance tasks automation is proposed here featuring a multi spectra machine-vision system based on the integrated use of infrared and visible light imaging supported by a tailored illumination system.

## 3. Experimental Setup

**Figure 4:**
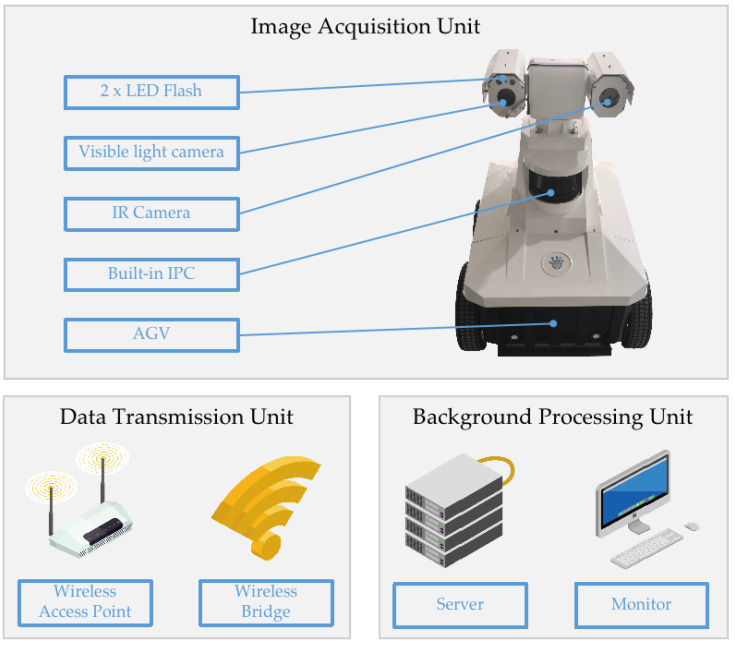
Inspection system hardware structure: the images acquired by the acquisition unit are sent to the processing unit through a wireless network endowed with an access point and a number of wireless bridges to cover the entire area. Images are then processed and stored in a dedicated server. A GUI visible on a monitor allows for interactive monitoring.

Taking into account the characteristics of weak illumination and complex environment in an actual industrial scene, this paper proposes a machine vision-based pipe leakage detection system. Such a system consists of three units: an image acquisition unit, wireless transmission unit, and background processing unit (as shown in Figure 4).

The image acquisition unit is composed of an automatic guided vehicle AGV (738 × 864 × 592 mm), visible light camera, two flash lights, an infrared camera, and an industrial personal computer (IPC). The visible light camera and infrared camera can capture high-definition images in two different light spectra at the same time [34]. In order to solve the problems of uneven light in the industrial environment and the consequent difficulty in identifying the features of colorless effusion, a pair of flash lights is designed for the image acquisition unit to expose the contour of different shapes of effusions more clearly.

The wireless transmission unit, made of an access point and a wireless network bridge, is responsible for establishing the wireless transmission channel from image acquisition unit to the data processing unit. The background processing unit includes hardware devices such as computing servers, computer monitor, and intelligent algorithms contained therein. The unit is used for image processing, data storage, and decision-making.

## 4. Inspection System Framework

**Figure 5:**
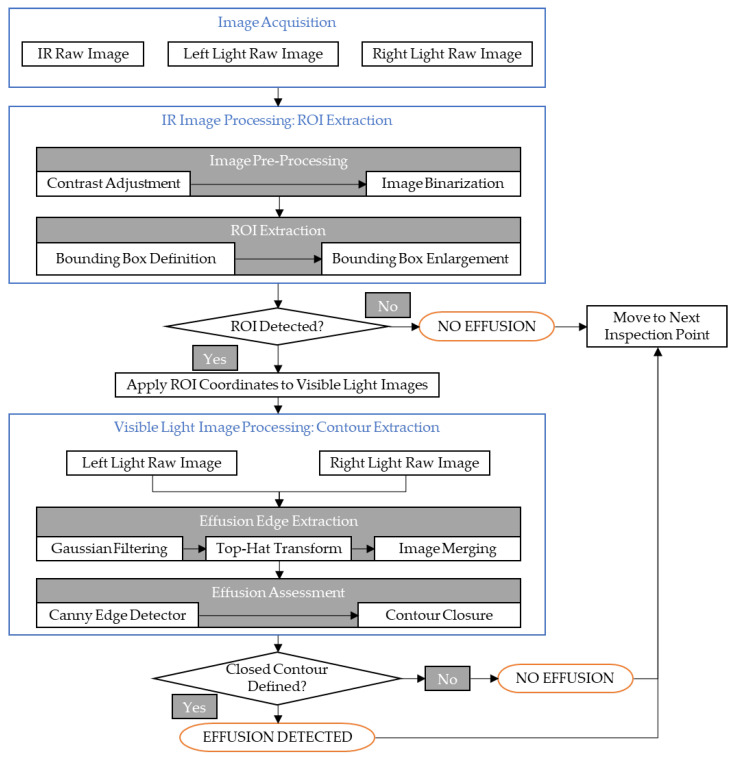
Detection system overall flowchart.

In order to overcome the interference and occlusions caused by complex background, this paper proposes an automatic inspection system for colorless effusion contour detection method based on the sequential analysis of multi-source images. The framework flow chart of the proposed system is reported in Figure 5.

The first step is the image acquisition, which consists in the simultaneous generation of three digital images, respectively consisting in an infrared image and two visible light images using a left and a right light source separately.

The second step involves the infrared image processing. In this respect, according to the low temperature characteristics of the effusion, the potential effusion is identified and its position is determined by the definition of a region of interest (ROI).

The third step is the visible light image processing in order to define the effusion contour. Then in the fourth step, the ‘top hat’ algorithm [35] is used to completely extract the effusion edge. The last step carries out a further image processing aimed at the effusion assessment enabled by the computation of the contour closure. 

To summarize, the overall process first uses infrared imaging to locate a potential effusion and then conducts a secondary assessment for the final effusion characterization. In this way, the leakage identification efficiency can be improved, and environmental interference can be reduced at the same time, thus improving the recognition accuracy.

With reference to Figure 5, the details of each single phase are explained in the remainder of this section.

### 4.1. ROI Extraction from Infrared Imaging

**Figure 6:**
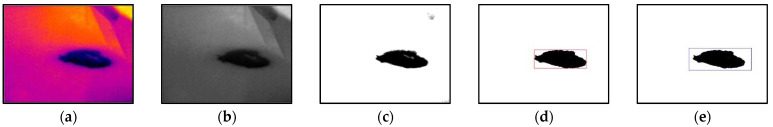
IR image processing procedure: (**a**) raw IR image in false colors, (**b**) infrared grayscale image, (**c**) enhanced contrast image, (**d**) bounding box, and (**e**) enlarged bounding box.

The temperature inside the power plant furnace reaches more than 1700 °C [36], requiring the use of side and wall air supply and other technologies to cool down the high temperature and avoid pipeline corrosion [37]. The temperature survey mentioned in Section 1 confirmed the temperature of effusion accumulation due to pipe water leakage is always lower than the ambient temperature, forming a temperature gradient in the pipeworks surrounding environment (as shown in Figure 1). Infrared thermal imaging technology allows for the surface temperature measurement of an object based on its thermal radiation energy [38]. In this respect, a potential leakage can be automatically identified by processing an infrared image, firstly performing a contrast enhancement, saturating the bottom 2% and the top 2% of all pixel values, and subsequently binarizing the image using Otsu’s thresholding method [39]. Such a technique has been selected due to its good binarization results with reference to the images under examination, the short computation time, and high implementation flexibility. From the binarized image, a region of interest (ROI) of the leakage area is defined by computing the bounding box of the resulting image region. In order to ensure that the ROI fully contains the complete edge contour of the leakage area, the rectangular bounding box is enlarged by 20% (as shown in Figure 6).

### 4.2. ROI Application to the Visible Light Imaging

**Figure 7:**
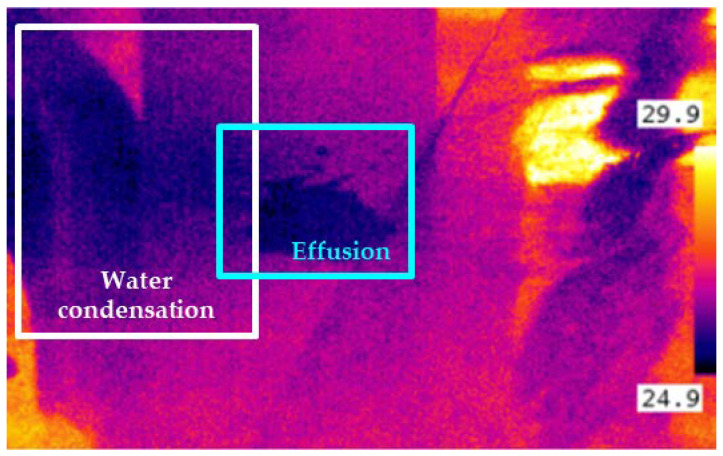
Infrared image of effusion in a humid environment, units in °C.

Although a colorless effusion has a lower temperature compared to the environment, the recognition of fluid accumulation within an infrared image could be potentially affected by water condensation phenomena on pipes, equipment, and floor. Figure 7 shows how, in a humid environment, water condensation and actual effusion could partially overlap, increasing the complexity of the classification task. This scenario indicates that, in the presence of humid environments, relying on the sole use of infrared imaging would produce a high false positive rate [30].

To overcome this issue, this paper combines the infrared imaging with visible light imaging. In this context, an infrared image is used to capture the potential effusion area. Subsequently, two visible light images are used to verify and assess the actual presence of the leakage/effusion as a classification task.

**Figure 8:**
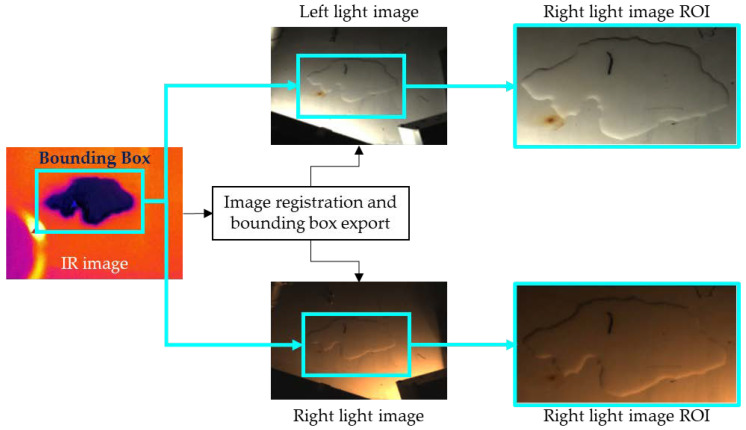
Visible light images ROI extraction process from the IR image. The ROI is firstly identified on the IR image, then the IR image and the visible light images are registered, and the ROI is exported and applied to the visible light images.

The specific process is as follows: for a single detection task, the system will acquire a set of three digital images (specifically an infrared image, a left light image, and a right light image). Initially the images need to be aligned to compensate the offset between the two cameras. Subsequently, an infrared image pre-processing is performed and aimed at the ROI extraction (as mentioned in Section 4.1). Then the computed ROI coordinates will be utilized on the left and right light source images, respectively (as shown in Figure 8).

### 4.3. Edge Extraction by Top-Hat Transform

**Figure 9:**
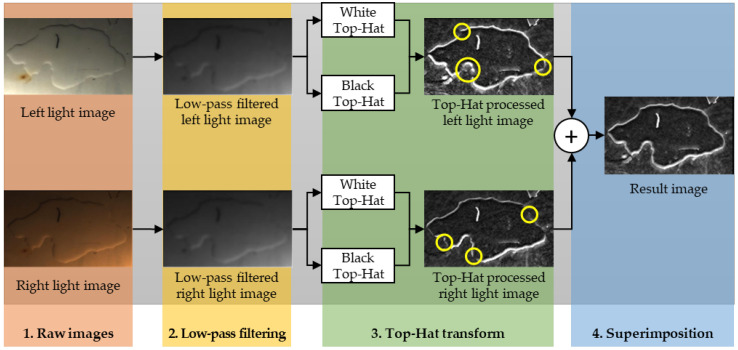
Visible image processing steps including gaussian denoising, top hat processing for contour extraction, and superimposition to remove contour discontinuities.

In this phase, the visible light raw images (Figure 9, step 1) are subject to a Wiener low-pass filtering [40] with a 5 × 5 pixel neighborhood size, with the aim of reducing the ambient noise. After filtering (as shown in Figure 9, step 2), the effusion contour presents a dark edge on the top (farther from the light source) and a bright edge on the bottom (closer to the light source). The effusion contour extraction is then carried out by applying a top-hat transform [35]. Specifically, a black top-hat and white top-hat transforms [35] were both applied to the input images and highlight the effusion contour.

A white top-hat (WHat) transform is an image processing procedure aimed at enhancing the areas with higher brightness in the image on the premise of maintaining the brightness distribution of the image [35]. Conversely, a black top-hat (BHat) transformation enhances the dark areas in the image under the constraint of maintaining the brightness distribution of the image [35]. 

In this paper, both WHat and BHat transforms have been configured with a 35 × 35 pixel neighborhood size and a diamond-shaped morphological structuring element [41] Following this twofold top-hat transform, both bright and dark edge features of the effusion are highlighted (as shown in Figure 9, step 3). 

Due to the light sources’ configuration and the water mass position, a single visible light source image can produce some discontinuities in the contour definition (see the yellow circles in Figure 9) leading to possible missing segments in the contour. To overcome this issue, the two top-hat processed images (i.e., left and right) were then superimposed to obtain the complete effusion contour characterization (Figure 9, step 4).

### 4.4. Effusion Detection by Canny Operator and Contour Closure Assessment

**Figure 10:**
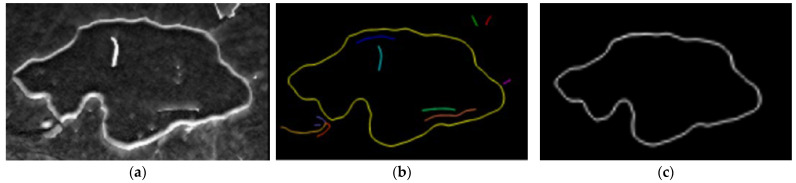
(**a**) Top hat processed image, (**b**) edge extraction results of effusion contour, and (**c**) result of effusion contour extraction.

The final stage of the effusion detection is carried out in this phase The images resulting from the previous steps are subject to a Canny operator-based [42] procedure to determine the effusion edge. This algorithm uses two different thresholds to detect two types of edges, namely strong and weak edges. Strong edges are defined as the actual effusion edges while the weak edges are potential noise which needs to be further assessed. The advantage of this algorithm is that it can identify the real strong and weak edge information in the image more accurately, highlight the edge information, and reduce the interference of the environment. The process result is shown in Figure 10.

The specific steps are as follows [43]: 

(1) The input image, shown in Figure 10a, is smoothed and denoised via Gaussian square filter, with a kernel size of 7 × 7 pixels.

(2) The gradient intensity and direction are computed for each pixel as per Equations (1) and (2).

(1) $$G=\sqrt{G_{x}^{2}+G_{y}^{2}}$$

(2) $$\theta =arctan\left(G_{y}/G_{x}\right)$$

where $G_{x}$ and $G_{y}$ are, respectively, the edge horizontal and vertical first derivatives values.

(3) An edge thinning procedure based on non-maximum suppression is applied. In this respect, a generic pixel is considered an edge point if its gradient intensity is the largest compared to the intensities of the two adjacent pixels along the positive and negative gradient directions. Otherwise, it is discarded.

(4) Two thresholds, namely high and low threshold, respectively, are defined to assess the strength of the edges. In order to be classified as a strong edge, the gradient intensity of a pixel must be higher than the high threshold. A gradient intensity value included between the two thresholds identifies a pixel as weak edge. A pixel is eventually discarded if its gradient intensity is lower than the low threshold.

(5) According to the above four steps, pixels classified as strong edges are identified as edges, and then weak edge pixels need to be further confirmed. In this respect, the weak edge pixels connections are explored within an eight-neighborhood space. If in such neighborhood there is at least a strong edge pixel, the weak edge point can be confirmed as a real edge. In this way, it is possible to completely map the effusion edges, as shown in Figure 10. In Figure 10b, the identified edges are represented in different colors. Specifically, the yellow line represents the contour features of the effusion, while the other colored lines are just noise which is then removed (as shown in Figure 10c).

After having detected the complete effusion contour, the Moore-neighbor tracing (MNT) algorithm was used to assess its closure. MNT finds the next contour pixel using eight connected chain codes with a clockwise sequence scanning [20,22]. Starting from a predefined pixel, if such a pixel is visited for a second time during the above-mentioned scanning, the contour is defined as closed, representing a real water effusion. Otherwise, the contour is defined as open, representing spurious water masses and noise.

## 5. Experimental Tests

**Figure 11:**
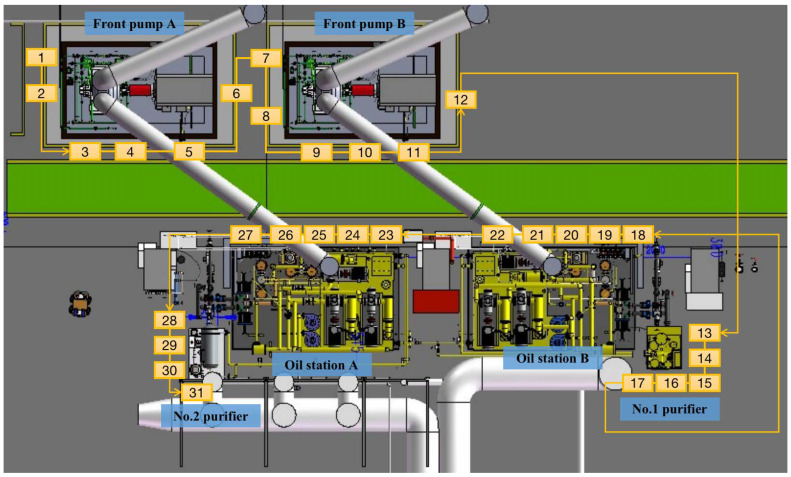
Top view of the overall inspection area including the equipment to be inspected, the 31 inspection points, and the inspection path.

With reference to Figure 11, the overall power plant floor is divided into six inspection areas based on the working equipment location: Area 1 (front pump A); Area 2 (front pump B); Area 3 (No.2 purifier device); Area 4 (oil station A); Area 5 (oil station B); and Area 6 (No.1 purifier device). Each inspection area includes a number of inspection points (i.e., critical points for equipment maintenance). There are 31 total inspection points distributed in 6 inspection areas. In this context, the AGV moves along a predefined inspection path covering the 31 inspection points, as illustrated in Figure 11. To each inspection point corresponds a specific AGV positioning location which is designed to provide for a clear view of the inspection point, minimizing the cluttering and shading.

**Figure 12:**
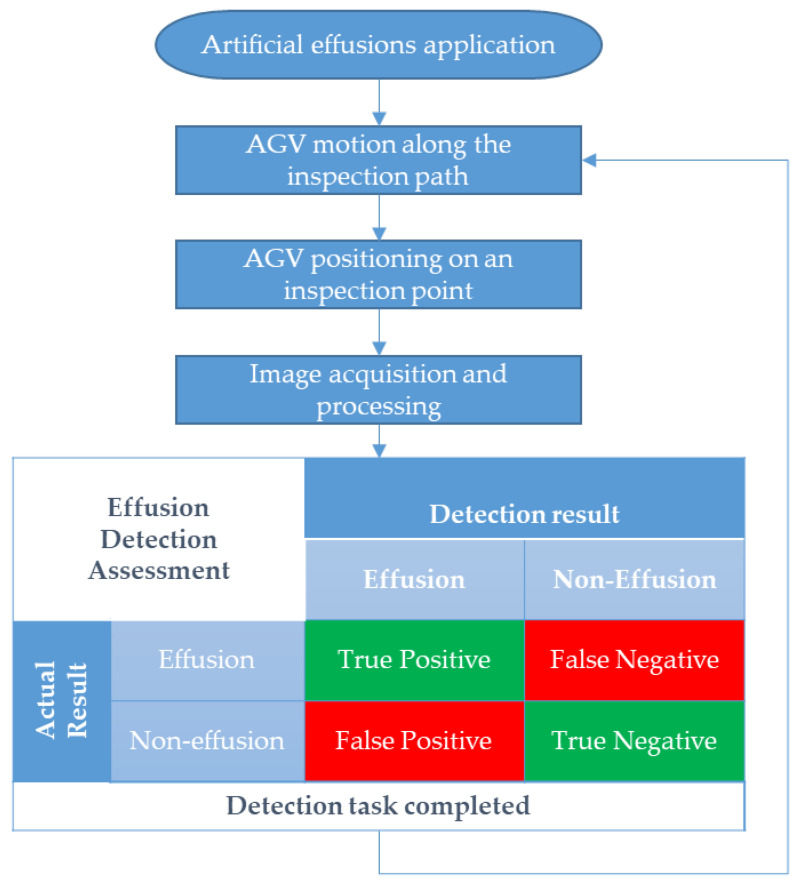
Flowchart of the experimental process including the effusion detection steps and assessment confusion matrix.

Figure 12 illustrates the experimental tests flow chart. Artificial effusions were placed manually by using 0.4 liters of tap water which, considering the floor material, generates an effusion surface of approximately 200 cm^2^. A total number of 461 effusions were placed for the experimental program.

The AGV moves along the inspection path and reaches an inspection point. Here, the system performs the image acquisition and processing algorithm described in Section 4.

The inspection task in these experimental tests consists in a classification problem in correspondence to each inspection point. If the system correctly detects an effusion, the test result is recorded as a true positive. Alternatively, if the system detects an effusion where there is none, the test result is recorded as a false positive. Conversely, if the system does not detect any effusion where actually there is none, the test result is reported as a true negative, whereas a missed effusion detection is reported as a false negative.

Following the classification task, the AGV moves to the next inspection point. Each inspection point was tested from 34 to 54 times to increase the statistical reliability of the experimental tests. In this way, a total number of 1367 tests were carried out.

## 6. Results and Discussion

Table 1 reports the experimental tests results for each inspection area, inspection points, and inspection samples in terms of misclassified instances and misclassification rate. Results are then summarized in the confusion matrix reported in Table 2.

Out of 1367 experimental tests, 12 samples were misclassified, yielding a total detection error rate of 0.88%. Six false positives instances were recorded: false detection on 2_5 due to the protection cover; false detection (twice) on 5_4 due to flashlight failure; false detection on 6_3 due to strong light interference; and false detection (twice) on 4_2 due to light changes.

The misclassification can be attributed to the system sensitivity which does not allow for the detection of small effusions which create water masses too small or too scattered to be detected. This issue can be tackled by acquiring higher resolution images and by performing periodical inspection rounds, allowing the water to form a detectable effusion. 

Analogously, effusions larger than the camera field of view are likely to be misclassified and, hence, not detected. In this case, specific wide-angle lenses should be utilized and the image processing procedure should be re-calibrated.

Moreover, the cluttering effect of complex equipment (such as pipework), although minimized, still badly affects the classification performances. In this respect, a more agile image acquisition system should be developed in order to acquire digital images in difficult-to-reach areas. Additionally, a denser inspection path could be designed by increasing the number of inspection points.

However, it is worth to highlight that the experimental setup designed in this paper, including the size of the tested effusions, has been defined based on the industrial experience, guidelines and requirements provided by the industrial partner.

## 7. Conclusions

The experimental results show that the proposed method can deploy the reflective and low temperature characteristics of water effusion for an accurate detection system which has the potential to boost the automation of the maintenance operations. In this paper, the potential water effusion area is preliminary identified via infrared imaging and further assessed by the subsequent method of water effusion identification based on the reflective and shape characteristics of the water mass in the corresponding area. Such an approach can effectively identify leakage water in the actual environment of a thermal power plant.

The overall classification success rate of the method proposed in this paper is above 99%. The experimental results show excellent suitability for the implementation in complex environmental conditions to detect the leakage water of different degrees. Other advantages include the detection capabilities of early micro-leakage water phenomena, strong environmental adaptability, and wide application range.

Future research efforts should be focused on strengthening the optimal path planning for AGV inspection path optimization, as well as the optimal design of environmental inspection points for the whole plant (along with the distribution of station points). In this respect, equipment endowed with multiple pipes scanning will be divided into central and secondary pipes for which tailored customized inspection paths should be defined with different schedules to optimize the system coverage along with the inspection and maintenance costs.

## Figures and Tables

**Table 1:** Experimental results.

Detection Area	Inspection Point ID	Inspection Samples	Misclassified Samples	Misclassification Rate (%)
Area 1 (Front pump A)	1_1	42	0	0.00
	1_2	46	0	0.00
	1_3	46	0	0.00
	1_4	46	1	2.17
	1_5	35	0	0.00
	1_6	42	0	0.00
Area 2 (Front pump B)	2_1	52	1	1.92
	2_2	44	0	0.00
	2_3	46	0	0.00
	2_4	52	0	0.00
	2_5	54	1	1.85
	2_6	44	0	0.00
Area 3 (No.2 purifier device)	3_1	40	0	0.00
	3_2	46	1	2.17
	3_3	42	0	0.00
	3_4	42	1	2.38
Area 4 (oil station A)	4_1	42	0	0.00
	4_2	44	2	4.55
	4_3	42	0	0.00
	4_4	42	1	2.38
	4_5	50	0	0.00
Area 5 (Oil station B)	5_1	42	0	0.00
	5_2	34	1	2.94
	5_3	40	0	0.00
	5_4	40	2	5.00
	5_5	50	0	0.00
Area 6 (No.1 purifier device)	6_1	46	0	0.00
	6_2	50	0	0.00
	6_3	44	1	2.27
	6_4	44	0	0.00
	6_5	38	0	0.00

**Table 2:** Detection accuracy confusion matrix.

Effusion Detection Assessment		Detection Result	
		Effusion	Non-Effusion
**Actual result**	**Effusion**	455	6
	**Non-Effusion**	6	900
